# A systematic review of the factors associated with the course of borderline personality disorder symptoms in adolescence

**DOI:** 10.1186/s40479-021-00151-z

**Published:** 2021-04-19

**Authors:** Gabriele Skabeikyte, Rasa Barkauskiene

**Affiliations:** Department of Clinical Psychology, Institute of Psychology, Faculty of Philosophy, Vilnius University, Universiteto st. 9, LT-01513 Vilnius, Lithuania

**Keywords:** Borderline personality disorder, Adolescence, Developmental trajectories

## Abstract

**Background:**

Research on personality pathology in adolescence has accelerated during the last decade. Among all of the personality disorders, there is strong support for the validity of borderline personality disorder (BPD) diagnosis in adolescence with comparable stability as seen in adulthood. Researchers have put much effort in the analysis of the developmental pathways and etiology of the disorder and currently are relocating their attention to the identification of the possible risk factors associated with the course of BPD symptoms during adolescence. The risk profile provided in previous systematic reviews did not address the possible development and course of BPD features across time. Having this in mind, the purpose of this systematic review is to identify the factors that are associated with the course of BPD symptoms during adolescence.

**Methods:**

Electronic databases were systematically searched for prospective longitudinal studies with at least two assessments of BPD as an outcome of the examined risk factors. A total number of 14 articles from the period of almost 40 years were identified as fitting the eligibility criteria.

**Conclusions:**

Factors associated with the course of BPD symptoms include childhood temperament, comorbid psychopathology, and current interpersonal experiences. The current review adds up to the knowledge base about factors that are associated with the persistence or worsening of BPD symptoms in adolescence, describing the factors congruent to different developmental periods.

## Background

Adolescence is a sensitive period for various psychological disturbances, including personality pathology [1]. During normative development, children’s maladaptive personality traits (such as emotional instability, neuroticism) tend to decline with age [2, 3]. However, there is a part of adolescents who diverge from the norm and whose personality problems tend to persist or even increase as adolescents enter young adulthood [1]. During the last decades researchers interested in adolescent personality pathology have mostly explored borderline personality disorder (BPD) which is characterized by turbulent interpersonal relationships, emotional instability, and an unstable sense of self [4]. Rejecting the hypothesis about adolescents’ difficulties only as a “storm and stress” period, there is strong support for the validity of a personality disorder (PD) diagnosis in adolescence with similar rank order stability in adolescents when compared with these features dynamics in adulthood [5, 6].

Personality disturbance does not simply manifest in adulthood, thus, research exploring the developmental precursors in young people with elevated personality disturbance create an opportunity to understand specific vulnerabilities and prodromal features, which may later turn into the emergence of a clinical disorder [7–9]. This notion is especially significant in adolescence when personality disorder is emerging and can be diagnosed in its early stage, but borderline symptoms are still flexible, making this developmental period an advantageous stage to intervene [10]. Furthermore, unrecognized borderline pathology during this developmental period has the potential to derail developmental achievements and disrupt the transition to adulthood [11–14].

Research on personality disorders in adolescence have started to accelerate during the last decade. While much effort has been put into the analysis of the etiology of BPD, scientists offer two important research directions: firstly, research must include repeated assessment of BPD during developmentally sensitive windows that may capture the course of the disorder in periods of peak prevalence [15]. Secondly, Chanen et al. (2017) offered that public health research priorities should be allocated in a way that the data would build up a knowledge base which would help to understand the risk factors for the persistence or worsening of problems, rather than the onset of the disorder itself [10].

Existing systematic reviews mainly focus on the examination of risk factors associated with the emergence or current mean levels of BPD symptoms and identify factors crossing multiple domains (e.g. social, family, maltreatment, child characteristics) [15–18]. However, they are lacking data about the course of already existing symptoms and factors that might contribute to the increases or decreases in BPD symptoms during adolescence. Moreover, most of the studies include adolescent as well as adult samples in their analysis which does not allow to capture risk factors specifically relevant to adolescence [15–17]. Based on the shortcomings arising from previous reviews, the purpose of the current systematic review is to identify the factors that are associated with the course of borderline personality disorder symptoms during adolescence.

## Methods

This systematic review was conducted using Preferred Reporting Items for Systematic Reviews and Meta-analyses (PRISMA) guidelines. The protocol was registered with PROSPERO in April of 2019 (registration no. CRD42019130158).

### Inclusion and exclusion criteria

To identify studies for inclusion, the following electronic databases were systematically searched: MEDline, PubMed, PsycINFO, PsycARTICLES, socINDEX, Proquest and Scopus. Search terms from which all possible variations were searched are listed in Table 1. Studies were limited to peer-reviewed articles written in English language and published from January of 1980 until March of 2020.

**Table 1:** Search terms used in the electronic database search

Key word	Search terms
Borderline personality disorder	Borderline personality disorder OR Borderline states OR Borderline personality symptoms OR Borderline personality features OR Borderline personality features OR BPD OR Borderline
Prospective	Longitudinal OR trajectory* OR prospective OR course OR “time point*” OR follow-up OR “Follow up”
Risk factors	“Risk factor “OR mechan* OR predict* OR precursor OR prodrom OR antecedent OR pathway OR interact* OR “protective factor” OR protective OR moderat* OR mediat*
Adolescence	Adolescence OR adolescents OR adolescent development OR adolescent psychopathology OR teens OR youth

Research methodology was based on the lacking theoretical aspects and limitations from the previous reviews: 1) Only prospective based longitudinal studies with a minimum of two time point intervals were included since previous reviews mostly evaluated the predictors of the mean levels of BPD, but failed to capture the actual change of BPD symptoms across time. 2) Research studies that describe only aspects of borderline personality disorder (e.g. self-harm, identity), but do not cover the entity of symptoms characterizing the clinical disorder were excluded as well as intervention studies. Studies that longitudinally assessed borderline personality symptoms as a dependent variable without the analysis of associated factors were excluded. Studies were included if they examined borderline personality symptoms or features as an outcome of the study. 3) In accordance with recent data indicating the importance of the extended developmental period from puberty to emerging adulthood for the early recognition of BPD [11], the study participants were adolescents aged 10 to 18 years old or adolescents as part of a ‘youth’ sample (e.g. 15–25 years old). Children under age 10 and adults older than 18 years of age, except for those who were part of the youth sample described previously, were excluded.

### Selection of articles

**Fig. 1:**
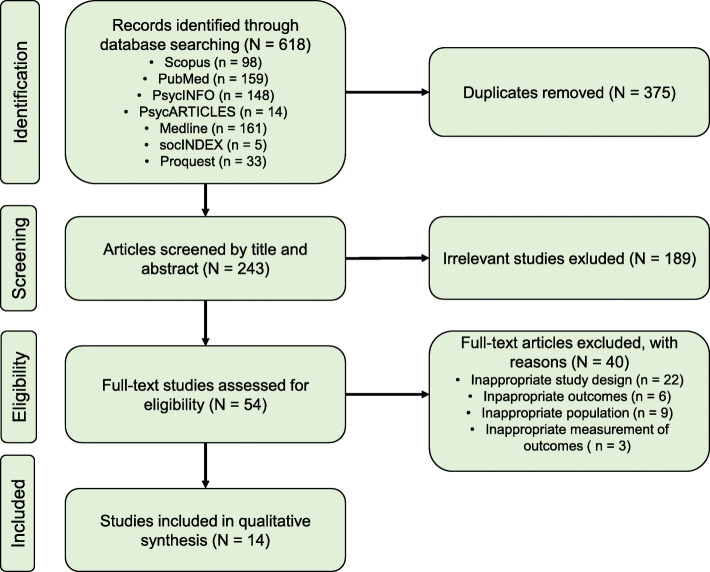
PRISMA diagram showing study selection process

Search results were transferred to a web-based tool “Covidence” which is designed for primary screening and data extraction (Cochrane, 2015). A total of 618 articles were identified through a database search. First of all, 375 duplicates were found and removed, leaving 243 articles for screening by title and abstract. Out of all studies, 189 did not meet the eligibility criteria for the analysis. After a full-text analysis by two reviewers, 40 studies were excluded on the basis of inappropriate study design, outcomes, measurement methods, or population. At each step, disagreements were resolved through a discussion and if necessary, a third reviewer helped to find a solution. A total of 14 studies, which provided longitudinal data about BPD symptoms and related features across adolescence, were included in the final analysis. Search results were summarized in a PRISMA chart (Fig. 1).

At the next step, the quality of the selected studies was assessed using the Quality Assessment Tool for Observational Cohort and Cross-Sectional Studies (National Health Institute, 2014). Two reviewers conducted independent assessments and overall quality ratings were categorized through a discussion as ‘good’, ‘fair’ “or ‘poor’ (*see* Table 2). Out of all studies, nine of them were rated as ‘good’ and five – ‘fair’. No studies were rated as poor, indicating an overall sufficient quality of the selected articles.

**Table 2:** Summary of risk of bias within studies

Author (date)	ResearchQuestion	Population	Participationrate	Recruitment	Sample sizejustification	Exposure prioroutcome	Time-frame	ExposureLevels	ExposureMeasure	RepeatedExposureAssessment	OutcomeMeasures	Blinding	Followup loss< 20%	StatisticalAnalysis	Qualityrating
Barnow et al. (2013) [19]	Y	Y	Y	Y	NR	Y	Y	Y	Y	N	Y	NA	Y	Y	FAIR
Bornovalova et al. (2018) [20]	Y	Y	Y	N	NR	Y	Y	Y	Y	Y	Y	NA	Y	Y	GOOD
Dixon-Gordon. et al. (2016) [21]	Y	Y	Y	Y	NR	Y	Y	Y	Y	N	Y	NA	Y	Y	GOOD
Ehrenreich et al. (2016) [22]	Y	Y	Y	Y	NR	Y	Y	Y	Y	Y	N	NA	N	Y	FAIR
Greenfield et al. (2015) [23]	Y	Y	Y	Y	NR	Y	Y	Y	CD	Y	Y	NA	N	N	FAIR
Hallquist et al. (2015) [24]	Y	Y	Y	Y	NR	Y	Y	Y	Y	Y	Y	NA	NR	Y	GOOD
Haltigan & Vaillancourt, (2016) [25]	Y	Y	CD	Y	NR	Y	Y	Y	Y	Y	Y	NA	N	Y	GOOD
Lazarus et al. (2019) [26]	Y	Y	Y	Y	NR	Y	Y	Y	Y	Y	Y	NA	Y	Y	GOOD
Sharp et al. (2020) [27]	Y	Y	Y	Y	NR	Y	Y	Y	Y	N	Y	NA	N	Y	GOOD
Stepp, Keenan, Hipwell & Krueger (2014) [28]	Y	Y	Y	Y	NR	Y	Y	Y	Y	Y	Y	NA	CD	Y	GOOD
Stepp et al. (2014) [6]	Y	Y	Y	Y	NR	Y	N	Y	N	N	Y	NA	CD	Y	FAIR
Stepp & Lazarus (2017) [29]	Y	Y	Y	Y	NR	Y	N	Y	Y	N	Y	NA	Y	Y	GOOD
Strandholm et al. (2017) [30]	Y	Y	Y	Y	NR	Y	Y	Y	N	Y	N	NA	Y	Y	FAIR
Vanwoerden et al. (2019) [31]	Y	Y	Y	Y	NR	Y	Y	Y	N	Y	Y	NA	N	Y	GOOD

*Y* yes, *N* no, *CD* cannot determine, *NR* not reported, *NA* not applicable

### Description of studies

A total of 14 studies were identified as appropriate for inclusion in further analysis. Key ideas from the articles were extracted and categorized by two reviewers. The following categories were described: study details (authors, year, country), study design, population (clinical or community), sample characteristics (sex, age range, sample size), sociodemographic data and outcome assessment methods. The main characteristics of the included studies are presented in Table 3.

**Table 3:** Characteristics of included studies

Author (year)	Country	Study design,BPD assessments	Population	Sample characteristics	Sociodemographic data	Outcome assessment
Barnow et al. (2013) [19]	Germany	Longitudinal,2 assessments	Community, Greifswaldfamily study	*N* = 381; range 11–18;55.1% female	NR	Structured Clinical Interview forDSM-III-R (SCID-II)1 and SCIDII-forDSM-IV1,2
Bornovalova et al. (2018) [20]	U.S.	Longitudinalcohort study,3 assessments	Community, Minnesotatwin family study	*N* = 1.080; range 14–24;100% female	95.3% white	Minnesota Borderline PersonalityDisorder Scale (MBPD)1
Dixon-Gordon et al. (2016) [21]	U.S.	Longitudinal,4 assessments	Community, Pittsburghgirls study	*N* = 113; range 16–18;100% female	Low-income neighborhoods; 65%African American, 35% White; 55%of familes receive public assisstance	Structured Clinical Interview forDSM-IV Personality Disorders(SIDP-IV)2
Ehrenreich, Beron &Underwood (2016) [22]	U.S.	Longitudinal,2 assessments	Community	*N* = 287; range 14–19;52% female	23.1% African American, 1.6% Asian,61.6% Caucasian, 18.3% Hispanic,5.4.% other	The Mclean Screening Instrumentfor BPD (MSI)1
Greenfield et al. (2015) [23]	Canada	Longitudinal,2 assessments	(In)Outpatient	*N* = 286; range 12–18;72% female	69.5% Caucasian, 7.5.% AfricanAmerican, 2.2.% Hispanic, 3.1%Aboriginal, 5.3.% Asian, 12.4% other	Abbreviated Diagnostic Interviewfor Borderlines (Ab-DIB)1
Hallquist, Hipwell & Stepp(2015) [24]	U.S.	Longitudinal,4 assessments	Community, Pittsburghgirls study	*N* = 2.228; range 14–17;100% female	Low-income neighborhoods	International Personality DisorderExamination-Screen (IPDE-S)1
Haltigan & Vaillancourt(2016) [25]	Canada	Longitudinal,4 assessments	Community, McMasterteen study	*N* = 566; range 13–16;55.5% female	NR	Borderline Personality Features Scalefor Children (BPFS-C)1
Lazarus et al. (2019) [26]	U.S.	Longitudinal,5 assessments	Community, Pittsburghgirls study	*N* = 2.310; range 15–19,100% female	Low-income neighborhoods; 59.8%Black, 40.2% White; 33.2% of familiesreceive public ssisstance	International Personality DisorderExamination (IPDE-BOR)1
Sharp et al. (2020) [27]	U.S.	Longitudinal,5 assessments	Community	*N* = 1.042; range 13–18;56% female	31.4% Hispanic, 29.4% White, 27.9%African Americans, 3.6% Asian, 7.7%other; 19.4% reveived mental healthtreatment	Borderline Personality Features Scalefor Children (BPFS-C)1
Stepp, Keenan, Hipwell &Krueger (2014) [28]	U.S.	Longitudinal,6 assessments	Community, Pittsburghgirls study	*N* = 2.282; range 14–19;100% female	Low-income neighborhoods; 53%African American, 41.2% EuropeanAmerican, 5.8% other;	International Personality DisordersExamination (IPDEBOR)1
Stepp et al. (2014) [6]	U.S.	Longitudinal,4 assessments	Community, Pittsburghgirls study	*N* = 2.212; range 14–17;100% female	38.9% of families receive publicassisstance	International Personality DisordersExamination (IPDEBOR)1
Stepp & Lazarus (2017) [29]	U.S.	Longitudinal,9 assessments	Community, Pittsburghgirls study	*N* = 2.344; range 14–22;100% female	Low-income neighborhoods; 53%African American, 41.2% Caucasian,5.8% other	International Personality DisordersExamination (IPDEBOR)1
Strandholm et al. (2017) [30]	Finland	Longitudinal,2 assessments	Outpatient, Adolescentdepression study	*N* = 218; range 13–19;81.5% female	Low-income neighborhoods; 58.7%minority race; 38.9% of familiesreceive public assisstance	Structured Clinical Interview andScreen (Personality Questionnaire)for DSM-IV PDs1,2
Vanwoerden, Leavitt, Gallagher& Temple (2019) [31]	U.S.	Longitudinal,5 assessments	Community	*N* = 818; range 16–21;58% female	32% Hispanic, 31.3% White, 27.1%African American, 1.8% Asian, 7.7.%other	Borderline Personality FeaturesScale for Children (BPFS-C)1

1 self-report instrument; 2 clinical interview; *NR* not reported

Out of all studies, ten of them were conducted in the U.S., two in Canada, one in Finland, and one in Germany. Six studies were based on the same study population, however, they analysed different aspects of the topic. Duration of the studies ranged from one to ten years, and population in the studies ranged from 113 to 2344 participants at baseline assessment. In seven studies females formed a full sample, two study samples were formed of 70–80% females, while in five other studies participants were more equally distributed by gender, with girls constituting 52–58% of the sample. Participants’ age ranged from 10 to 24 years of age. Twelve studies were based on community samples and two on (in) outpatient samples. Outcomes of the studies mostly were measured by self-rating scales of borderline personality disorder symptoms, except three studies that included structured clinical interviews for the assessment of BPD symptoms. All of the methods used in the studies were based on the DSM-IV or ICD-10 symptom-oriented approach towards personality disorders.

### Main results of the current review

The results revealed a large heterogeneity of the studies in terms of the reported analyses of BPD symptoms, course, domains of the associated factors, and their timing as predictors. First, in line with the previous research on normative personality development [2, 5], authors of the majority of the studies (10 of 14) report data about the general decreasing trajectory of BPD symptoms during adolescence which was seen both in the community and in the clinical samples. However, there is a part of youth who deviate from the normative developmental trajectory and fall into the persisting BPD symptoms group in the clinical sample (76% of adolescents) [23] and into the elevated/rising (24% of adolescents; 74% girls) or intermediate/stable BPD symptoms groups (42% of adolescents; 54% girls) in the community sample [25]. Second, as the purpose of this review suggests, only factors that were longitudinally associated with increases or decreases in the mean levels of BPD symptoms as an outcome, will be included. Presented studies will further be categorized based on the domain of the associated factors that were examined. The detailed classification of the analysed factors is presented in Table 4.

**Table 4:** The classification of the analysed factors based on the factor domain and study sample

Author (year)	Study sample	Child characteristics	Interpersonal factors	Parental psychopathology	Parenting factors	Covariates
Barnow et al. (2013) [19]	Greifswald family study^a^	–	–	Maternal BPD symptoms,maternal depression	–	Sex, age, BPD features in offspringsat T_0_
Bornovalova et al. (2018) [20]	Minnesota twin family study^a^	Alcohol use disorder, druguse disorder, major depressivedisorder	–	–	–	NR
Ehrenreich, Beron & Underwood(2016) [22]	Community^a^	Social and physical aggression	–	–	–	Baseline ratings of rule-breaking,internalizing symptoms; borderlinefeatures and narcissism at Grade 7
Haltigan & Vaillancourt (2016) [25]	McMaster teen study^a^	Temperament, somatization,ADHD symptoms, anxiety,depression, general academicfunctioning	Peer victimization,relational aggression	–	–	Sex, mental health, peer relations,intra-individual risks
Sharp et al. (2020) [27]	Adolescent dating violencestudy^a^	Lifetime mental health treatment	Parent-child relationshipquality	–	Exposure to intimatepartner violence	Sex, minority status, familycomposition/living situation, mentalhealth treatment history, parenteducation, relationship qualitywith each parent
Vanwoerden, Leavitt, Gallagher &Temple (2019) [31]	Adolescent dating violencestudy^a^	–	Psychological violence,sexual violence, physicalviolence, relational violence	–	–	Sex, SES, relationship quality witheach parent
Dixon-Gordon et al. (2016) [21]	Pittsburgh girls study^a^	Negative affect	–	–	Maternal problemsolving, maternalsupport/validation	Minority race, family poverty
Hallquist, Hipwell & Stepp (2015) [24]	Pittsburgh girls study^a^	Negative emotionality, harshpunishment, self-control	–	–	–	Previous ratings of harsh punishment,self-control, negative emotionality
Lazarus et al. (2019) [26]	Pittsburgh girls study^a^	–	Perceived support,antagonism, physicalaggression, verbalaggression	–	–	Minority race, family poverty, pubertaldevelopment
Stepp, Keenan, Hipwell & Krueger(2014) [28]	Pittsburgh girls study^a^	Negative emotionality, highactivity, low sociability, lowshyness	–	–	–	Minority race, family poverty
Stepp et al. (2014) [6]	Pittsburgh girls study^a^	Impulsivity, negative affectivity,ODD/CD severity	–	Parental depressionseverity	Harsh punishment,low warmth	Minority race, family poverty
Stepp & Lazarus (2017) [29]	Pittsburgh girls study^a^	Emotionality, inattention,hyperactivity/impulsivity, depression	–	–	–	Minority race, family poverty
Greenfield et al. (2015) [23]	(In) Outpatient, previouslysuicidal adolescents^b^	Age of suicidal behavior, depression,conduct disorder, alcohol use, druguse, overall severity of disturbance,stressful life events, emergency roomvisits, hospitalizations	–	–	–	Sex, age
Strandholm et al. (2017) [30]	Outpatient with depressivemood disorders, Adolescentdepression study^b^	Depression severity, comorbidity	Social support fromfamily and friends	–	–	Sex, age, SSRI medication, number ofclinical appointments during thefollow-up

^a^ community sample; ^b^ clinical sample;

*NR* not reported

### Child characteristics

The most examined domain of the factors associated with the course of BPD symptoms during adolescence was child characteristics. To start with, temperament dimensions, such as high levels of emotionality, activity and low levels of sociability and shyness in middle childhood were predictive of higher elevations as well as increases in average levels of BPD features through adolescence [28]. In contrast, negative affectivity assessed in early and middle adolescence was only predictive of higher mean levels of BPD [6], but not anymore of the change in these features over time [21]. Moreover, the data further suggest that the link between negative affectivity in early adolescence and increases in the mean levels of BPD features from middle adolescence is not a direct one, but rather mediated by decreases in self-control skills [24].

Among other child-related factors, the authors also have evaluated the role of stressful life events (suspension from school, death of a parent, changes in peer acceptance, etc.) at ages 12–17 in the clinical sample, but did not found statistically significant associations [23]. In the community sample, general academic functioning measured by the standardized assessment procedure at age 8 was not statistically predictive of changes in BPD features during adolescence [25].

Adolescent psychopathology as a predictor of BPD symptom changes was analysed in eight of the fourteen studies. Within the community samples, it was found that childhood psychopathology, such as inattention, oppositional behaviour, and hyperactivity/impulsivity predicted the change to the new onset status of BPD in adolescence [29]. In line with previous findings, impulsivity and oppositional defiant disorder severity assessed in adolescence were also associated with higher average levels of BPD symptoms throughout adolescence [21]. Furthermore, it was identified that alcohol use disorder (AUD), drug use disorder (DUD), major depressive disorder (MDD) symptoms [20], anxiety symptoms, attention deficit hyperactivity disorder (ADHD) symptoms and somatization [25] statistically significantly predicted the changes in BPD features during adolescence. Specifically, higher average levels and increases in AUD, DUD, and MDD symptoms were associated with a slower decline of BPD symptoms through adolescence [20]. Adolescent-reported symptoms of ADHD and somatization also predicted the elevated or rising symptom trajectory, while parent-reported anxiety levels predicted stable intermediate levels of BPD features [25]. Moreover, individual social and physical aggression trajectories from childhood through adolescence were not significantly related to the BPD symptoms change from age 14 to 18 [22].

Results from two clinical samples mostly capture child-related psychopathology factors. Firstly, in line with the findings from the community sample, decreases in depression severity and comorbidity were associated with faster declines in average levels of BPD symptoms [30]. Secondly, lower levels of a child’s general psychosocial functioning was statistically predictive of BPD clinical diagnosis at follow-up 4 years later [23].

### Interpersonal factors

Interpersonal factors in relation to BPD symptom dynamics were examined in six of the fourteen studies. Several important relationship-based factors were found to be significant as predictors of changes in BPD features in adolescence. First of all, studies show that the experience of relational aggression in the context of friendship is predictive of the elevated or rising BPD symptoms trajectory [25]. In addition, psychological and sexual violence [31] as well as perceived support and antagonism [26] in romantic relationships are predictive of increases in the mean levels of BPD features over time. Physical and verbal aggression experienced within romantic relationships were not predictive of BPD feature change or average levels [26]. Moreover, relationship quality with the father predicted slower declines in BPD features through adolescence [27]. In the analysed clinical samples, family relations, social support from friends and family were not statistically significantly associated with changes in BPD symptoms [23, 30].

### Parental psychopathology

Two studies provide data about several important parental psychopathology factors assessed in adolescence: maternal BPD symptoms, maternal depression [19], and parental depression severity [6]. Studies failed to detect statistically significant BPD symptom associations with parental psychopathology, except maternal BPD symptoms. It was found that only maternal BPD characterized by six or more symptoms constitutes a risk for higher average BPD levels in the offspring at follow-up 5 years later [19]. In these studies, parental depression severity was not associated with changes in BPD symptoms [6, 19].

### Parenting factors

Analyses of parenting practices have revealed that in adolescence, parental low warmth [6], maternal support/validation, and maternal problem solving [21], average levels or changes in parental harsh punishment [6, 24] were not significant predictors of changes in BPD features. Among parenting factors, exposure to intimate partner violence among parents was the only factor associated with BPD symptom changes and predicted slower declines in BPD symptoms throughout adolescence [27].

## Discussion and limitations

The purpose of this systematic review was to identify the factors that are associated with the course of BPD symptoms during adolescence. Fourteen studies were identified as corresponding to the inclusion criteria and have provided significant data about the associated factors which might contribute to the course of adolescent BPD symptoms.

First of all, although the declining BPD features trajectory was seen in the majority of the analysed studies, researchers have identified a group of adolescents whose BPD symptoms or features were persisting or even increasing during adolescence [23, 25]. These results go in line with Sharp et al. (2018) notion about normative declines in maladaptive personality traits and increases in the groups where these features are significantly prominent [1]. Stability of symptoms or increases were seen both in the clinical and in the community samples, which reveals that there is a part of youth with difficulties in personality development not only in the clinical setting, but also in the community sample.

In context of the analysed studies, findings suggest that individual and interpersonal domains of functioning stand out as accommodating the majority of factors significantly associated with changes in BPD symptoms through adolescence. From the individual perspective, several childhood and adolescent psychopathology conditions which prevent the normative decline of maladaptive personality traits during adolescence and predict changes in BPD features were identified. To start with, externalizing psychopathology in childhood statistically significantly predicted the change of BPD features in adolescent girls [29]. In addition, difficult childhood temperament [28, 29] and poor self-control [24] were associated with the increasing BPD features trajectory. Alongside childhood maladjustment, adolescence-related psychopathology that was associated with changes in BPD symptoms was marked by a variety of difficulties and included substance use disorders, major depressive disorder [20], ADHD symptoms, somatization [29] as well as comorbidities in general [30]. Since BPD has high comorbidity rates [1, 4], it is not surprising that changes in the comorbid states affect the trajectory of BPD features. Bornovalova et al. (2018) explain these results using a pathoplasty model which reveals that symptoms of comorbid states disrupt maturational processes and contribute to the persistence or worsening of BPD [20]. Sharp, Vanwoerden & Wall (2018) have concluded that personality disorders are preceded by childhood internalizing and externalizing disorders [1], however, results of the current review reveal that they might continue to shape the developmental trajectory of BPD symptoms in adolescence. From a clinical standpoint, these findings denote the importance of the on-time recognition of externalizing and internalizing problems and intervention as early as possible to block the way for a full-blown BPD and its further development during adolescence.

Another important domain was interpersonal factors which reflect current relational experiences. It was found that being exposed to peer-related violence in friendships and in romantic relationships is associated with increases in BPD symptoms across time. These experiences include relational, psychological, and sexual violence as well as antagonism as a bidirectional behaviour [25, 26, 31]. Adolescence is an important period in the context of learning to create and maintain relationships [32] and in this way damaging interpersonal behaviours may disrupt the process of normal personality development. Moreover, it is worth to mention that not only disruptive interpersonal behaviour, but also experiences incompatible with normative development, such as excessive reliance on or perceived support from a romantic partner in intense early romantic relationships, also were associated with increases in girls BPD symptoms [26]. When considering the importance of family relations, it was found that poorer relationship quality with the father prevents the normative decline in BPD features over time [27]. Overall, the results reveal the great significance of negative experiences in current relationships on the course of BPD symptoms during adolescence. They also indicate the need for more comprehensive assessments of the factors analysing adolescents’ social relations in future studies on adolescents’ personality pathology.

Furthermore, much effort has been put in the analysis of parenting and parental psychopathology factors since parental neglect, emotional under involvement, or invalidation appear to contribute to the development of BPD [15, 33]. However, the only parenting-related factor that was associated with changes in BPD symptoms was the exposure to interparental intimate partner violence, conceptualized as physical aggression [27]. This reflects the greater importance of the family environment and social interactions being observed, but not the parenting behaviours themselves. Other parenting factors that were previously presented were not significant in predicting changes in BPD features [6, 21, 24]. Authors consider that parenting factors perhaps are more important in the earlier developmental stages or in their capacity to predict the onset of the disorder, not changes in symptoms across time [24]. Moreover, there is strong evidence for the greater role of peer relationships in adolescence compared with familial ones. According to Harmelen et al. (2017), when controlled for the effects of family support, only friendship support may predict later resilient psychosocial functioning and may serve as a strong protective factor in adolescence [34].

Comparing the results from the clinical and community-based samples, we may see that factors associated with changes in personality pathology are partially overlapping in both groups. However, studies with clinical samples were focused on the role of comorbid psychopathology [23, 30] and stressful life events [23] rather than interpersonal factors that have been found to be significant predictors in high risk and community samples [25, 26, 31]. Based on the existing results so far, we can conclude that only comorbid psychopathology was found as a joint predictor of change in BPD features both in the clinical and in the community samples of adolescents. However, the study quality ratings have revealed some methodological drawbacks in two clinical studies, which means that the results must be considered carefully. To sum up, more longitudinal studies with clinical samples are needed in order to better understand the distinction or similarities between the community and the clinical risk profiles. Reflecting on the implications for the further research we want to note that the risk profile from each study is more representative of a specific domain of functioning (e.g. psychopathology) without taking into account other possible factors. None of the analysed studies included several domains of factors which could potentially address the complex nature of the processes related to the course of personality pathology during adolescence.

From a clinical perspective, developmental staging model suggests that identifying a group of adolescents with specific risk factors or subthreshold symptoms is necessary for the on-time intervention [35]. Our review suggests that an adolescent who would demonstrate a risk of getting on the increasing BPD trajectory would be one with difficult temperament dimensions brought from childhood, having comorbid states, and currently experiencing victimization from peers or exposure to violence at home. Chanen et al. (2016) also elaborates on the importance of comorbid mood disorders in the transition from the mild or subthreshold symptom stage to the onset of the disorder [35]. This risk profile corresponds to the recent review by Hutsebaut & Aleva (2020) where they have also proved the importance of the associated mental disorders and current interpersonal context in predicting the severity of BPD in both adolescents and adults. Extending our results, adverse childhood experiences, BPD symptom severity, and personality traits were also reported as significant factors for poor BPD prognosis [16], however, they have not been investigated in longitudinal studies as predictors of changes. In fact, factors that were delineated by Hutsebaut and Aleva (2020) and associated with the poor BPD prognosis [16], could possibly also affect changes in BPD symptoms throughout adolescence. In general, previous systematic reviews [15–18] represent the data about the risk factors associated with the mean levels of BPD features through a lifespan and mostly include individual and parental factors. This review extends the scope about the importance of factors associated with peer-relationships. Therefore, the results of the current review add up to the knowledge base about factors that are specifically associated with the persistence or worsening of BPD features which can already be seen in adolescence and cover the factors congruent to the current developmental period as well as those from middle childhood.

The conclusions based on the results from this systematic review should be interpreted in the light of the number of limitations. First of all, six of the analysed studies were drawn from the same sample which was formed only of urban girls, and have provided the results about childhood psychopathology and temperament. Hence, there is a potential risk for bias in our interpretation and the significance of effects. Moreover, studies lacked consistency in the measurement of BPD symptoms, since a variety of BPD measurement methods (including different self-report scales and interviews) were used. However, during the quality assessment of each study, 12 out of 14 studies were rated as providing clearly defined and valid outcome measures with decent psychometric properties. In addition, multiple informants (adolescents, parents, teachers) provided information about associated risk factors. In line with different methodologies, several studies provided different conceptualizations of the same terms, e.g., drug use was conceptualized as a clinical syndrome [20] or as a delinquent behaviour [23] which could explain the contradictory results. In addition, despite that we have excluded intervention studies, participants in the clinical samples might have been provided with intervention between the assessments. Future research directions could be allocated to analyse the course of BPD symptoms in a more diverse and gender-balanced sample and would include factors that could capture different domains of functioning.

## Conclusions

Clinicians and researchers agree that BPD should become a novel public health priority since it has high personal and community costs [10]. This systematic review has revealed that comorbidity may play an important role in the course of borderline personality disorder development as well as current interpersonal experiences. However, the risk profile suggested by this review is not a unique one, nor the final. Future research should accumulate data on other potentially important factors and their interactions in predicting the course of BPD in adolescence, which would help to create a more precise profile of adolescents at risk [15, 16].

## Data Availability

Not applicable.
